# Long non-coding RNA LUCAT1 inhibits myocardial oxidative stress and apoptosis after myocardial infarction via targeting microRNA-181a-5p

**DOI:** 10.1080/21655979.2021.1966351

**Published:** 2021-08-20

**Authors:** Shi-Hui Xiao, Ying Wang, Xuecai Cao, Zhe Su

**Affiliations:** a Department of Internal Medicine-Cardiovascular, Ganzhou People’s Hospital, Ganzhou, Jiangxi Province, China; b Xi’an Jiaotong University Health Science Center, Xi’an, Shaanxi Province, China; c Department of Cardiology, Affiliated Hospital of Gansu Medical College, Pingliang, Gansu Province, China; d Department of Obstetrics, Yidu Central Hospital of Weifang, Weifang, Shandong Province, China; e Department of Emergency Internal Medicine, The Affiliated Hospital of Qingdao University, Qingdao, Shandong Province, China

**Keywords:** LUCAT1, miR-181a-5p, acute myocardial infarction, apoptotic, oxidative stress

## Abstract

This study hoped to explore the effects and mechanism of long non-coding RNA (lncRNA) LUCAT1 regulating microRNA-181a-5p (miR-181a-5p) on oxidative stress and apoptosis of cardiomyocytes induced by H_2_O_2_. Totally, 72 patients with acute myocardial infarction (AMI) were included. H9c2 cardiomyocytes were cultured in vitro, and the H_2_O_2_ model of cardiomyocytes was established. The expression levels of LUCAT1 and miR-181a-5p were detected by qRT-PCR after H_2_O_2_ induction. The contents of reactive oxygen species (ROS), superoxide dismutase (SOD), and malondialdehyde (MDA) in cells were detected. The survival rate of the cells was detected by the Cell Counting Kit-8 (CCK-8) method; the apoptosis was detected by flow cytometry. The luciferase reporter experiment and quantitative real-time PCR (qRT-PCR) were used to verify the targeted relationship between LUCAT1 and miR-181a-5p. LUCAT1 was lowly expressed in the AMI patients. After H_2_O_2_ induction, the expression of LUCAT1 in H9c2 cells lessened significantly, while the expression of miR-181a-5p elevated significantly (*P* < 0.001). Transfection of p-LUCAT1 significantly reversed the decreased SOD levels, the increased MDA and ROS content, and the elevated tumor necrosis factor-alpha (TNF-α), interleukin-6 (IL-6), and interleukin-1 beta (IL-1β) in H_2_O_2_-stimulated cells (*P* < 0.001). Upregulation of LUCAT1 contributed to the mitigation of H_2_O_2_ injury by promoting viable cells and repressing apoptotic cells (*P* < 0.01). LUCAT1 targeted miR-181a-5p and negatively regulated miR-181a-5p expression (*P* < 0.001). Collectively, LUCAT1 played a protective role on oxidative stress injury, inflammation, viability, and apoptosis of cardiomyocytes induced by H_2_O_2_ via regulating miR-181a-5p.

## Introduction

Acute myocardial infarction (AMI) is a cardiovascular disease with high morbidity and mortality [1]. Coronary artery occlusion caused by unstable plaque rupture and erosion secondary thrombosis is the main characteristic of AMI [2]. Due to the insufficient blood and oxygen supply from the coronary artery, the main clinical manifestation of patients is persistent and severe pain behind the sternum, which poses a serious threat to the patient’s health and even life safety [3,4]. In recent years, with the establishment of chest pain centers in various regions as well as the progress of thrombolytic therapy and stent implantation, the mortality of patients with AMI has been significantly reduced [5]. However, the prognosis of patients with AMI is affected because there is no effective treatment to improve oxidative stress and inflammation in the progression of AMI [6]. Therefore, it is of great value to study the pathological process of AMI injury in order to reduce the poor outcomes of patients with AMI.

Long non-coding RNA (lncRNA) is an endogenous cellular RNA, which is an mRNA-like transcript and varies in length from 200 nt to 100 kb [7]. LncRNAs cannot be used as a template for the synthesis of protein, but several lncRNAs have been identified to play important roles in many cardiovascular diseases [8]. For example, lncRNA SNHG8 can regulate myocardial infarction and may become a potential target of myocardial infarction [9]. LINC00528 is lowly expressed in the cell models, and it can repress the cell apoptosis and viability by regulating microRNA-143-3p (miR-143-3p) and further controlling the levels of COX-2 [10]. The expression of HOTAIR is enhanced in the H9c2 cell models caused by H_2_O_2_, and HOTAIR promotes cell viability and restricts inflammation of myocardial cells via sponging miR-126 [11]. Association of miR-93 and lncRNA MORT is identified in the cardiomyocytes, and the contrary functions are proposed in the regulation of cell apoptosis [12]. However, the role and mechanism of abnormal expression of lncRNA LUCAT1 in myocardial injury after AMI are still unclear. There is more and more evidence that LUCAT1 is one of the genetic risk factors of cardiovascular diseases, including coronary heart disease and cardiomyopathy, which suggests that LUCAT1 may also be a potential genetic marker of AMI [13]. In addition, silencing of LUCAT1 is approved in chronic heart failure, and it can function as a beneficial role in the development of heart failure by accommodating cell viability and apoptosis via miR-612, suggesting that LUCAT1 plays a promoting role in myocardial injury [14].

In this study, we aimed to explore the expression of LUCAT1 in AMI and investigate the potential mechanism of LUCAT1 in oxidative stress, inflammation, viability, and apoptosis of cardiomyocytes. We discussed the function of LUCAT1 in the occurrence and development of AMI, systematically studied the expression and role of LUCAT1 in cardiomyocytes under H_2_O_2_, and explored its target gene to provide an experimental and theoretical basis for finding new molecular markers for the detection and treatment of AMI.

## Materials and methods

### Patients and sample collection

A total of 72 patients with AMI were selected from the Affiliated Hospital of Qingdao University. At the same time, 67 volunteers who underwent physical examination in our hospital were selected. Five milliliters of peripheral venous blood from the above-selected patients were collected. All patients were examined for the first time and never received any treatment or surgery aiming at AMI before.

### RNA extraction and quantitative real-time PCR (qRT-PCR)

Total RNA was extracted with TRIzol reagent and reverse transcribed according to a previous study [15]. The sequences of LUCAT1, miR-181a-5p, GAPDH, and U6 were amplified by qRT-PCR, and the expression of LUCAT1 in each group was corrected with GAPDH as an internal reference. The total reaction system was 20 μl, which contains complementary DNA (cDNA, 50 ng) 2 μl, upstream and downstream primers (10 μM) 1 μl, TB Green Fast qPCR Mix (2×) 10 μl, and ddH_2_O 6 μl, respectively. The reaction conditions were as follows: pre-denaturation was carried out at 95°C for 5 min, then denatured at 95°C for 15 s, and annealed at 60°C for 30 s for 40 cycles. The relative expression of LUCAT1 and miR-181a-5p was calculated by formula 2-Ct. The experiment was carried out three times.

### H9c2 cell grouping and transfection

The experiment was divided into six groups: (1) blank control group, namely normal cultured H9c2 cells; (2) H_2_O_2_ group, cells of which were treated with H_2_O_2_ (concentration 100 μmol/L) for 4 h to establish oxidative injury model; (3) H_2_O_2_ + p-negative control (NC) group, cells of which were transfected by pCDNA3.1 carrying LUCAT1 NC; (4) H_2_O_2_ + p-LUCAT1 group, cells of which were transfected via pCDNA3.1 carrying LUCAT1 sequence; (5) H_2_O_2_ + si-NC group, cells of which were transfected using si-NC; and (6) H_2_O_2_ + si-LUCAT1 group, cells of which were transfected with si-LUCAT1. Lipofectamine 3000 (Invitrogen, Carlsbad, CA, USA) was used in transfection experiments. The treatment method of H_2_O_2_ was based on a previous publication [16].

### Important parameter detection

The concentration of indicators relative to oxidative stress, including reactive oxygen species (ROS), superoxide dismutase (SOD), and malondialdehyde (MDA) in cells, was detected by separate kits. Besides, inflammatory cytokines were also assessed by ELISA kits. All these experiments were conducted in accordance with a reference [17].

### Cell viability assay

Cell viability was detected by the Cell Counting Kit-8 (CCK-8) method via referring to a previous investigation [18]. The experimental cells were treated with 0.25% trypsin and seeded into each well of a 96-well plate. Ten microliters of CCK-8 reagent (Genomeditech, Shanghai, China) were added to each well and further incubated for 2 hours. The OD value of each well was detected under 450 nm of a microplate reader.

### Cell apoptosis assay

Cell apoptosis assay of H9c2 cells was assessed by flow cytometry [19]. The cells in the logarithmic growth phase were inoculated in a 6-well plate and then washed twice with phosphate buffer solution and centrifuged to discard the supernatant. About 195 μl binding buffer was added to the collected cell precipitation and cell suspension was obtained. Then, 5 μl Annexin V-FITC and 10 μl propidium iodide reagent were added, and the plate was placed in a cool and lightless condition for 20 min. After that, flow cytometry was performed to detect apoptotic cells .

### Luciferase activity assay

The luciferase activity was assessed as per a previous report [10]. MiR-181a-5p mimics, miR-181a-5p inhibitors, and relative NC were from purchased GenePharma (Shanghai, China). The mutation of LUCAT1 and wide LUCAT1 was obtained and cloned into pCDNA3.1 vectors separately. A co-transfection experiment was carried out to transfect different plasmids together with the previous three miR-181a-5p sequences. After 48-h transfection, cells were collected, and a reaction between cells and luciferase was performed. Then, intensities of firefly luciferase (Luc) and Renilla luciferase (Rluc) were detected, respectively.

### Statistical analysis

The results of clinical parameters of AMI patients were statistically analyzed by SPSS software. Others were analyzed by the GraphPad software. All results were expressed as a number or mean ± standard deviation. Independent-sample *t*-test, chi-square test, and single-factor analysis of variance were used for multi-group mean comparison (*P* < 0.05).

## Results

In the present study, we aimed to detect the levels of LUCAT in AMI patients and explore the underlying mechanism behind LUCAT1. For this purpose, we conducted qRT-PCR to assess the expression of LUCAT1 in AMI patients. Furthermore, the impacts of LUCAT1 on oxidative stress, inflammation, viability, and apoptosis were explored in the H_2_O_2_-treated H9c2 cells. Besides, the ceRNA of LUCAT1 was confirmed.

### Clinical characters of two cohorts

In this study, the general data of all candidates were analyzed, and the results are shown in Table 1. There was no significant difference in gender and age groups (Table 1, *P* > 0.05). In order to exclude the influence of other factors on the relative expression of LUCAT1 in the trial group, the risk factors related to coronary heart disease were statistically analyzed, including the effects of hypertension, smoker, total cholesterol (TC), triglycerides (TG), low-density lipoprotein (LDL), high-density lipoprotein (HDL), and diabetes. The results suggest that there were no significant differences between the healthy people and AMI patients concerning clinical factors of AMI (Table 1, *P* > 0.05).

**Table 1.** Subjects’ basic clinical information

Parameter	Healthy individuals (*n* = 67)	AMI patients (*n* = 72)	*P*-value
Sex (male,%)	35, 52.2	34,47.2	0.554
Age (years)	56.96 ± 8.07	58.19 ± 9.87	0.421
Hypertension (No., %)	35, 52.2	44, 61.1	0.291
Smoker (No., %)	26, 38.8	35, 48.6	0.244
Diabetes (No., %)	19, 28.4	28, 38.9	0.190
TC (mmol/L)	4.12 ± 0.57	4.30 ± 0.56	0.068
TG (mmol/L)	1.32 ± 0.36	1.39 ± 0.37	0.239
LDL (mmol/L)	2.65 ± 0.41	2.74 ± 0.42	0.192
HDL (mmol/L)	1.27 ± 0.41	1.19 ± 0.34	0.243

TC, total cholesterol; TG, triglycerides; LDL, low-density lipoprotein; HDL, high-density lipoprotein.

### LUCAT1 was lowly expressed in AMI patients

**Figure 1.**
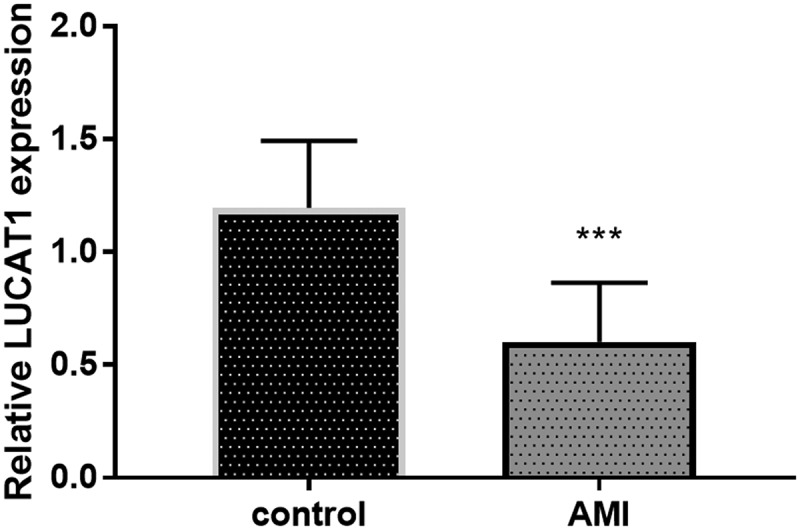
The expression of LUCAT1 was reduced in the AMI patients compared to control individuals. ***P < 0.001

The expression of LUCAT1 was identified in patients with AMI for the study of the effects of LUCAT1. Interestingly, reduced expression of LUCAT1 was provided by the results of qRT-PCR (Figure 1, *P* < 0.001). This finding proposed that the abnormally expressed LUCAT1 might be relative to AMI.

### P-LUCAT1 successfully reversed the influence of H_2_O_2_ on oxidative stress

**Figure 2.**
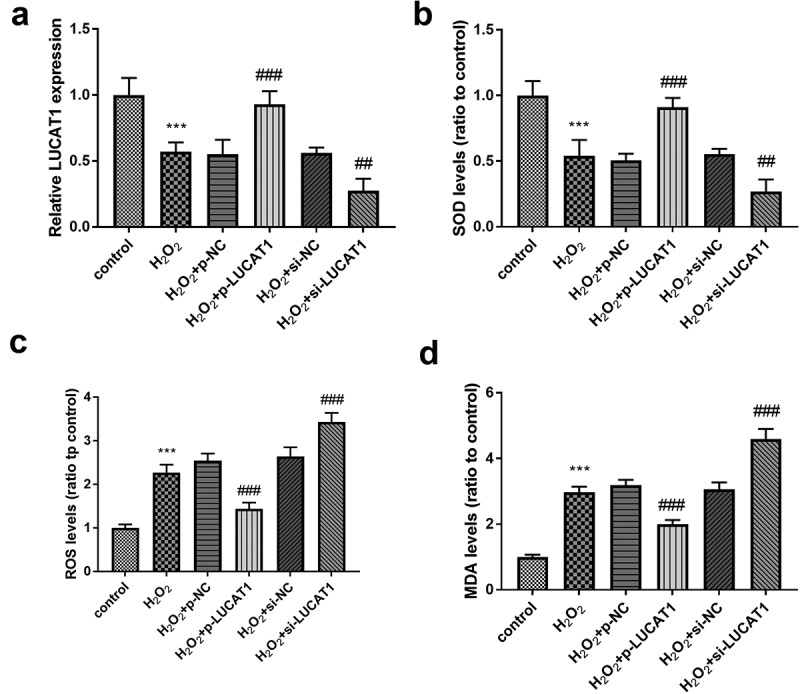
The impacts of LUCAT1 on H_2_O_2_-treated cells. (a) The transfection of p-LUCAT1 reversed the decreased LUCAT1 expression steered by H_2_O_2_ and the knockdown of LUCAT1 improved the function of H_2_O_2_. (b) The overexpression of LUCAT1 reversed the abnormally decreased SOD levels caused by H_2_O_2_, while the underexpression of LUCAT1 increased SOD levels caused by H2O2. (c) ROS levels were increased in the H_2_O_2_ group, which was further inhibited in the H_2_O_2_ + p-LUCAT1 group and improved in the H_2_O_2_ + si-LUCAT1 group. (d) The upregulation of LUCAT1 repressed the enhancement of MDA levels in the H_2_O_2_-treated cells and the downregulation of LUCAT1 promoted the enhancement of MDA levels. ***P < 0.001, compared with control group; ##*P* < 0.01, ###*P* < 0.001, compared with H_2_O_2_ group

Further functional research was analyzed in the cells stimulated by H_2_O_2_. The expression of LUCAT1 in the H_2_O_2_ group was significantly lower than that in the control group (Figure 2a, *p* < 0.001), which was consistent with the previous result. Compared with the H_2_O_2_ group, the expression of LUCAT1 was significantly upregulated in H_2_O_2_ + p-LUCAT1 group (Figure 2a, *p* < 0.001), which showed that p-LUCAT1 transfection was successful. The transfection of si-LUCAT1 successfully decreased the expression of LUCAT1 in the H_2_O_2_-treated cells (Figure 2a, *p* < 0.01).

SOD activity in H_2_O_2_-triggered cells decreased significantly, while ROS activity and MDA content increased significantly (Figure 2b-Figure 2d, *P* < 0.001). Compared to H_2_O_2_ groups, SOD activity in H_2_O_2_ + p-LUCAT1 group increased significantly and that in H_2_O_2_ + si-LUCAT1 group decreased significantly, indicating that LUCAT1 benefited the abundance of SOD levels (Figure 2b, *p* < 0.01). Moreover, the elevated LUCAT1 expression suppressed the raised levels of ROS and MDA under H_2_O_2_ circumstance and the silenced LUCAT1 had an opposite effect, indicating LUCAT1 might ameliorate the oxidative stress (Figure 2c-Figure 2d, *P* < 0.001).

### LUCAT1 protected H9c2 against and inflammation steered by H_2_O_2_

**Figure 3.**
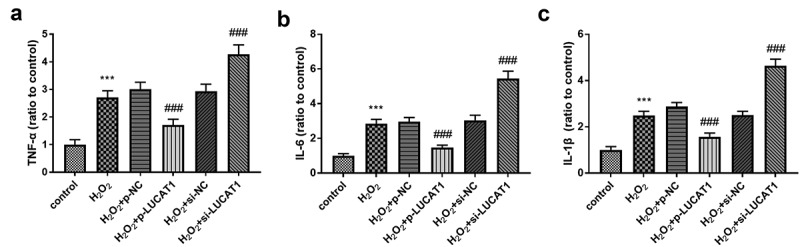
H_2_O_2_ led to the increased concentration of (a) TNF-α, (b) IL-6, and (c) IL-1β. Interference of LUCAT1 improved these trends, but LUCAT1 exerted inhibited functions on the secretion. ***P < 0.001, compared with control group; ###*P* < 0.001, compared with H_2_O_2_ group

The content of inflammatory activators was detected to study the influence of LUCAT1 on inflammation. As exhibited in Figure 3a, the tumor necrosis factor-alpha (TNF-α) was raised in the cells caused by H_2_O_2_, whereas LUCAT1 restricted inflammatory conditions by partially preventing the levels of TNF-α (*P* < 0.001). Additionally, the upregulation of LUCAT1 reduced the increased levels of interleukin-6 (IL-6) in the H_2_O_2_ group (Figure 3b, *p* < 0.001). The content of interleukin-1 beta (IL-1β) decreased significantly in H_2_O_2_-engendered cells, while this progression was ameliorated by the transfection of p-LUCAT1 (Figure 3c, *p* < 0.001). In addition, the interference of LUCAT1 improved the concentration of TNF-α, IL-6, and IL-1β in H_2_O_2_-managed cells (Figure 3a-Figure 3c, *P* < 0.001).

### Beneficial Influence of LUCAT1 on H9c2 cell models

**Figure 4.**
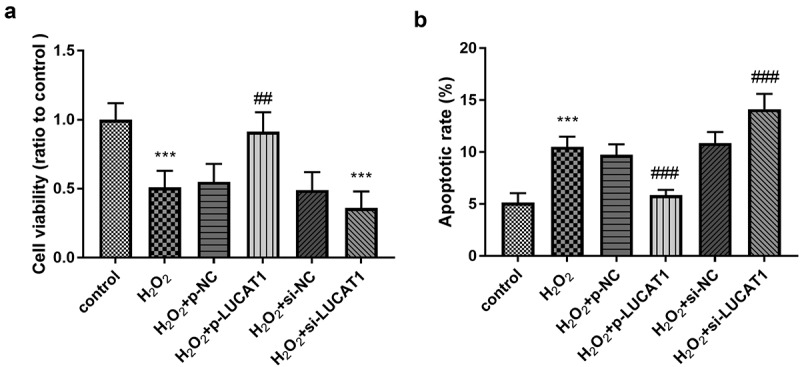
The function of LUCAT1 on cell viability and apoptosis. (a) The cell viability was suppressed in the H_2_O_2_ group, while overexpression of LUCAT1 reversed this trend and silenced LUCAT1 facilitated this trend. (b) The treatment of H_2_O_2_ facilitate the cell apoptosis, while increased expression of LUCAT1 meliorated this impact and decreased expression of LUCAT1 improved this impact. ***P < 0.001, compared with control group; ##*P* < 0.01, ###*P* < 0.001, compared with H_2_O_2_ group

To explore whether LUCAT1 could reverse the fate of cells stimulated by H_2_O_2_, the cell vitality was measured on H9c2. The results showed that the management of H_2_O_2_ could obviously destroy the cell viability, and CCK-8 results showed that LUCAT1 could significantly reduce the inhibition of cell proliferation irritated by H_2_O_2_ (Figure 4a, *p* < 0.01), suggesting that LUCAT1 participated in the amelioration of viability of H9c2 cardiomyocytes induced by H_2_O_2_. The silenced LUCAT1 inhibited the cell viability compared to the H_2_O_2_ group (Figure 4a, *p* < 0.001).

The apoptotic rate of H9c2 cells in the H_2_O_2_ group was significantly increased relative to the control group (Figure 4b, *p* < 0.001), suggesting H_2_O_2_ induced the injury of H9c2. Compared with the H_2_O_2_ group, the apoptotic H9c2 cells in the H_2_O_2_ + p-LUCAT1 group were significantly reduced and those in the H_2_O_2_ + si-LUCAT1 group were significantly raised (Figure 4b, *p* < 0.001), manifesting the possibility of the protective role of LUCAT1.

### MiR-181a-5p serves as a ceRNA of LUCAT1

**Figure 5.**
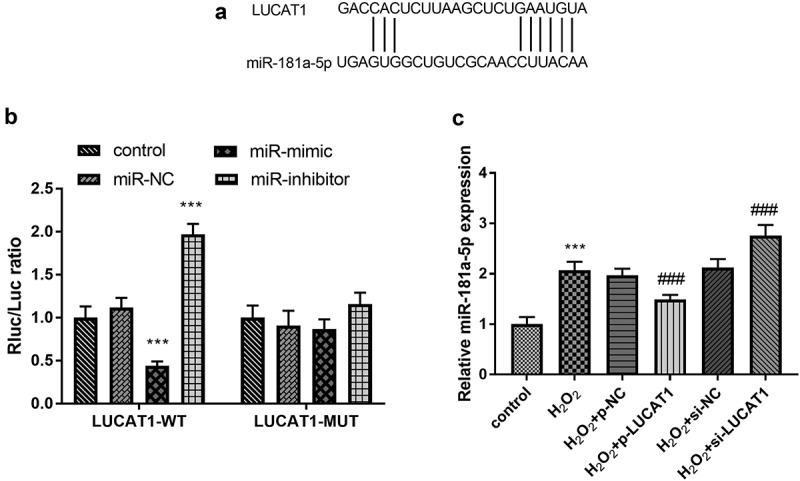
(a) The predictive binding sites between LUCAT1 and miR-181a-5p. (b) Overexpression of LUCAT1 inhibited the luciferase activity and underexpression of LUCAT1 elevated the luciferase activity in the LUCAT1-WT group. (c) Overexpression of LUCAT1 abrogated the upregulation of miR-181a-5p in the H_2_O_2_ group and inhibited expression of LUCAT1 accelerated the upregulation of miR-181a-5p in the H_2_O_2_ group. ***P < 0.001, compared with control group; ###*P* < 0.001, compared with H_2_O_2_ group

As shown in Figure 5a, there were binding sites between LUCAT1 and miR-181a-5p, indicating that LUCAT1 and miR-181a-5p might have a targeting relationship. The outcome of the luciferase reporting experiment validated that miR-181a-5p mimics could significantly reduce the relative luciferase activity of LUCAT1-WT cells (Figure 5b, *p* < 0.001), and no significant effect was found on the relative luciferase activity of LUCAT1-MUT cells (Figure 5b, *p* > 0.05). In addition, the treatment of H_2_O_2_ led to the enhancement of miR-181a-5p levels in H9c2 cells (Figure 5c, *p* < 0.001). In addition, the abundance of LUCAT1 restricted the aberrantly expressed miR-181a-5p and the intervention of LUCAT1 improved the expression of miR-181a-5p (Figure 5c, *p* < 0.001).

## Discussion

Arrhythmia and heart failure caused by ischemia–reperfusion injury will affect the curative effect of patients seriously [20]. How to take effective measures to reduce myocardial reperfusion injury and improve the prognosis of AMI patients is a hot and difficult point in clinical research [21]. Oxidative stress is involved in the pathogenesis of coronary heart disease, which is closely related to myocardial reperfusion injury [22]. Previous studies have found that free radical damage and inflammation are important mechanisms in the damage of ischemia–reperfusion [23]. This process can lead to the apoptosis and necrosis of myocardial cells, but it also provides a new target for clinical treatment [24]. In view of the importance of early diagnosis and treatment for AMI, researchers have turned their attention to the exploration of new therapeutic targets for AMI patients to a greater extent.

In the current investigation, we found that on the condition of non-differences on clinical parameters between the control group and AMI patients, the relative expression of LUCAT1 was obviously decreased in the patients with AMI, when compared to the healthy people, which indicates that the change of LUCAT1 expression might be a consequence of the development of AMI. The momentousness of lncRNAs in the continuance of angiocardiopathy has been explored in a multitude of researches. Pan finds the elevated expression of lncRNA H19 in patients with atherosclerosis, and it may aggravate the damage of atherosclerosis by managing the ability of proliferation and apoptosis [25]. Another publication explores the change of lncRNA ANRIL in patients with coronary heart disease, which indicates that ANRIL is lowly expressed in patients and may serve as an independent indicator [26]. The abnormal changes of LUCAT1 are also researched by lots of investigators. For example, the expression of LUCAT1 is aberrant in the tissues of patients with non-small-cell lung cancer or hepatocellular carcinoma [27,28]. Furthermore, LUCAT1 is downregulated in the patients with chronic heart failure, which ptovides an involvement between LUCAT1 and heart disease [29]. In addition, in a study published in 2020, the authors describe the expression of LINC00528 in cell models of myocardial infarction and explore its roles. In contrast to this study, our research showed the levels of LUCAT1 in AMI patients and explored its roles in four aspects, including oxidative stress, inflammation, viability, and apoptosis.

As oxidative stress and inflammation were the pathological progresses in the damage of AMI, the function of LUCAT1 on these two aspects was also detected. The cell models were constructed by H_2_O_2_ and the reduced expression of LUCAT1 was engendered by H_2_O_2_. The transfection of p-LUCAT1 successfully elevated the levels of LUCAT1 and transfection of si-LUCAT1 diminished the levels of LUCAT1, which provided a possibility of studying the impacts of LUCAT1. The findings on the SOD, ROS, and MDA substantiated that LUCAT1 exerted beneficial impacts on the amelioration of oxidative stress in H_2_O_2_-triggered H9c2 cells. Moreover, the inferior influence of H_2_O_2_ on inflammatory responses was reversed by the overexpression of LUCAT1 and enhanced by the underexpression of LUCAT1, elucidating that LUCAT1 might participate in the development of AMI by moderating the inflammatory situation. In a recent observation in 2020, LUCAT1 was identified as an inhibitor of inflammatory activity by repressing IL-6 [30]. The previous researches and prevent results indicated that LUCAT1 could mitigate the inflammation and oxidative stress induced by H_2_O_2_. The function of LUCAT1 on cell activities was also provided, and the outcome provided evidence that LUCAT1 ameliorated the injury of H_2_O_2_ on cell viability and apoptosis. In abdominal aortic aneurysm and pancreatic ductal adenocarcinoma, LUCAT1 also shows regulatory effects on cell viability and apoptosis [31,32].

LncRNA plays the role of miRNA molecular sponge, which is an indispensable mechanism of its participation in the regulation of myocardial injury. In a publication of Liu et al., miR-181a-5p participates in the progression of inflammation in the bone marrow mesenchymal stem cell models of myocardial infarction, which attracts our attention [33]. In addition, Liu et al. indicate that miR-181a-5p acts as a ceRNA of LUCAT1 in breast cancer [34]. Thus, we detected the relationship between miR-181a-5p and LUCAT1. The luciferase report evidenced that miR-181a-5p was a ceRNA of LUCAT1. In the acute cellular rejection of heart transplantation, the expression of miR-181a-5p is obviously elevated [35]. Moreover, miR-181a-5p plays an essential role in the regulation of high glucose-managed cardiomyocytes [36]. Our exploration demonstrated that the miR-181a-5p has reduced expression in the H_2_O_2_-steered cells. In addition, this regulatory connection between LUCAT1 and miR-181a-5p was identified by the conclusion that overexpression of LUCAT1 reduced the expression of miR-181a-5p and downregulation of LUCAT1 elevated miR-181a-5p expression. In research from Liu et al., the expression of miR-181a-5p is elevated in cells under lipopolysaccharide and managed the inflammation [33]. One limitation of this investigation was the limited number of patients. A Lack of information about miR-181a-5p expression in AMI patients was also a limitation of our study.

To sum up, the loss of LUCAT1 expression was found in patients with AMI relative to the healthy controls. After H_2_O_2_ induction, the expression of LUCAT1 lessened significantly in H9c2 cells, and the expression of miR-181a-5p raised significantly. LUCAT1 could play a role in protecting cardiomyocytes from oxidative stress injury and inflammation induced by H_2_O_2_, which may effectively reduce apoptotic cardiomyocytes and facilitate cell viability by binding miR-181a-5p. Therefore, LUCAT1/miR-181a-5p molecular axis was expected to be a new target for preventing and treating injury in AMI.

## Conclusion

Collectively, we found that the expression of LUCAT1 was lessened in AMI patients. Overexpression of LUCAT1 had protective effects on AMI via inhibiting the influence of H_2_O_2_ on oxidative stress, inflammation, viability, and apoptosis of H9c2 cells. Besides, miR-181a-5p was a ceRNA of LUCAT1.

## Data Availability

**The data that support the findings of this study are available from the corresponding author upon reasonable request.**

## Highlights

1. The expression of LUCAT1 was diminished in AMI patients.

2. LUCAT1 ameliorated the impacts of H_2_O_2_ on oxidative stress,

3. LUCAT1 protected cells against H_2_O_2_ damage on inflammation.

4. LUCAT1 had protective effects on cell viability and apoptosis.

5. LUCAT1 might exert functions via binding miR-181a-5p.
